# Functional roles, regulatory mechanisms and theranostics applications of ncRNAs in alcohol use disorder

**DOI:** 10.7150/ijbs.81518

**Published:** 2023-02-21

**Authors:** Jie-quan Wang, Ya-ru Liu, Qing-rong Xia, Jun Liang, Jin-liang Wang, Jun Li

**Affiliations:** 1 Department of Pharmacy, Affiliated Psychological Hospital of Anhui Medical University, Hefei, 230000, China; 2 Department of Pharmacy, First Affiliated Hospital of Anhui Medical University, Hefei, 230022, China; 3 Department of Pharmacy, Hefei Fourth People's Hospital, Hefei, 230000, China; 4 Psychopharmacology Research Laboratory, Anhui Mental Health Center, Hefei, 230000, China; 5 Anhui Clinical Research Center for Mental Disorders, Hefei,230000, China; 6 The Grade 3 Pharmaceutical Chemistry Laboratory of State Administration of Traditional Chinese Medicine, Hefei, 230022, China; 7 Anhui Province Key Laboratory of Major Autoimmune Diseases, Anhui Institute of Innovative Drugs, School of Pharmacy, Anhui Medical University, Hefei, 230032, China

**Keywords:** alcohol use disorder, noncoding RNAs, emerging biotechnology, gene therapy agents, regulatory mechanisms, theranostics applications

## Abstract

Alcohol use disorder (AUD) is one of the most prevalent neuropsychological disorders worldwide, and its pathogenesis is convoluted and poorly understood. There is considerable evidence demonstrating significant associations between multiple heritable factors and the onset and progression of AUD. In recent years, a substantial body of research conducted by emerging biotechnologies has increasingly highlighted the crucial roles of noncoding RNAs (ncRNAs) in the pathophysiology of mental diseases. As in-depth understanding of ncRNAs and their mechanisms of action, they have emerged as prospective diagnostic indicators and preclinical therapeutic targets for a variety of psychiatric illness, including AUD. Of note, dysregulated expression of ncRNAs such as circRNAs, lncRNAs and miRNAs was routinely found in AUD individuals, and besides, exogenous regulation of partial ncRNAs has also been shown to be effective in ameliorating alcohol preference and excessive alcohol consumption. However, the exact molecular mechanism still remains elusive. Herein, we systematically summarized current knowledge regarding alterations in the expression of certain ncRNAs as well as their-mediated regulatory mechanisms in individuals with AUD. And finally, we detailedly reviewed the potential theranostics applications of gene therapy agents targeting ncRNAs in AUD mice. Overall, a deeper comprehension of functional roles and biological mechanisms of ncRNAs may make significant contributions to the accurate diagnosis and effective treatment of AUD.

## Introduction

Alcohol use disorder (AUD) is a kind of chronic recurrent psychiatric illness characterized by an increased tolerance to alcohol, compulsive drinking regardless of physical and mental health, and loss of control over alcohol intake, ultimately leading to the progressive emergence of withdrawal syndrome, grand mal seizures, Wernicke encephalopathy (WE) and even death 1. The consequences of AUD concerned considerable disability, comorbidity and mortality, as well as substantial medical, caregiver and socioeconomic burdens 2, 3. On account of the easy availability and rapidly increasing consumption of alcohol, AUD has become one of the most pervasive mental diseases globally, with a male to female ratio of approximately 5:1 4, 5. Epidemiological studies showed that the prevalence of AUD was highest in developed countries (8.4%, 95% CI 8.0-8.9), followed by middle-income countries (5.4%, 5.0-6.0), and lowest in less developed countries 1. Although tools such as the Diagnostic and Statistical Manual of Mental Disorders (DSM) and International Classification of Disease (ICD) criteria, questionnaires and blood tests played an indispensable role in the screening and diagnosis of AUD, less than 15% of AUD patients were diagnosed and treated in a timely manner 6. Owing to evident contraindications and poor therapeutic effect, the clinical application of anti-craving drugs and alcohol-sensitive drugs was extremely limited, accounting for a persistently high recurrent incidence of AUD 7. What's worse, the early diagnosis and treatment of AUD still face great challenges as the number of drinkers increases and the abstinence rate declines year by year 4.

AUD is a complicated multifactorial illness whose etiology and pathogenesis have yet been completely understood. It is estimated that 40-60% of AUD risk can be attributed to hereditary factors, with the remainder being linked to individual physiopsychological features, social contextual variables and gene-environment interactions 6. Long-lasting changes in the expression of brain protein-coding genes have been the most intensively studied in AUD patients and rodents 8, 9. However, these genes only accounted for less than 2% of the mammalian genome sequence, and the rest of genomic transcripts are noncoding RNAs (ncRNAs) that were previously assumed to be incapable of coding for proteins 10. More specifically, ncRNAs are a diverse class of bioactive regulatory molecules that are broadly present in organisms, mediating multiple biological processes such as chromatin and histone remodeling, mRNA splicing, transcription, translation, post-transcriptional modification and signal transduction 11. Moreover, it has recently been shown that ncRNAs possessed the hidden potential to encode peptides and proteins 12. Emerging biotechnologies such as next-generation sequencing, microarray, single cell RNA sequencing (scRNA-seq) and genome-wide association studies (GWAS) have outperformed traditional genetic techniques in terms of detection efficiency, accuracy and automation 13. In practice, a large number of studies based on cutting-edge biotechnologies have revealed that ncRNAs were abundant in central nervous system (CNS) and played a regulatory role in brain function and homeostasis, as well as the pathological processes of mental diseases through a range of genetic and epigenetic mechanisms 14, 15. As a consequence, it was hypothesized that the regulatory network in which ncRNAs participate may affect potential molecular targets and signaling pathways that drive specific cellular biological responses and fates, eventually leading to the occurrence and development of AUD.

**Figure 1:**
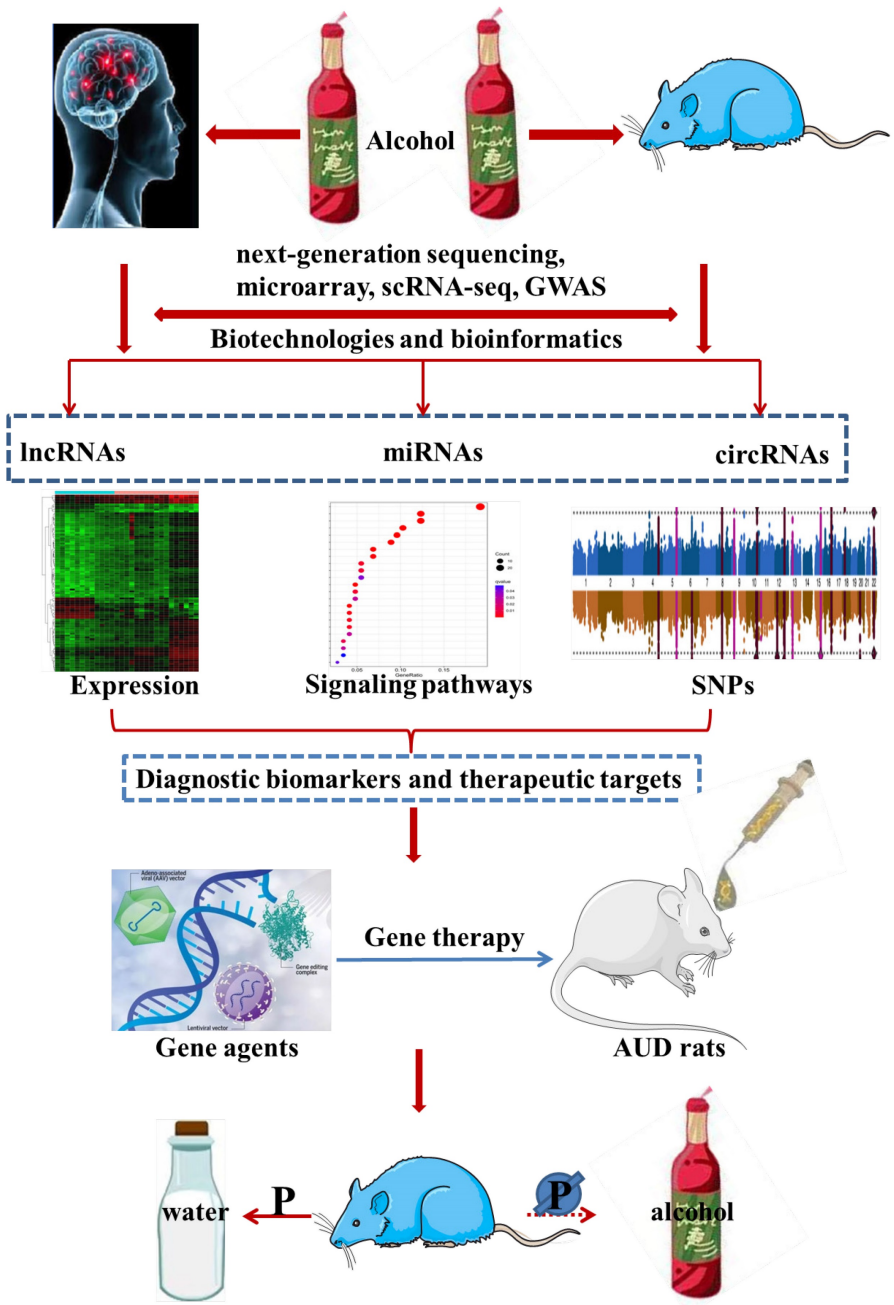
The content outline of our present review.

The current situation of AUD diagnosis and treatment is not encouraging, and the urgency to shed light on the etiology and pathophysiology of AUD is becoming more and more prominent. Notably, ncRNAs have been widely used as prospective biomarkers for many different types of diseases, and fortunately, considerable advance has been made in the treatment of a variety of disease models by simulating or silencing the AUD-related ncRNAs 16-19. More recently, a great deal of studies have assessed the expression of ncRNAs as well as their relevant molecular functions and biological mechanisms in AUD individuals. Despite many exciting advances, reliable diagnostic biomarkers for AUD and their relationship with AUD pathogenesis remain to be further explored. In this review, we mainly focused on the dysregulated expression of ncRNAs in AUD patients and animal models, and then, introduced their diagnostic value and prospects for future research and theranostics application. In addition, we systematically summarized current knowledge regarding the pathogenic ncRNAs-mediated biological mechanisms and functions in AUD individuals. Finally, we discussed the therapeutic effect of ncRNAs-targeting gene therapy agents on AUD animal models in detail. In summary, this review presented a comprehensive description of significant relationships between the expression and single nucleotide polymorphisms (SNPs) of ncRNAs and the progression of AUD, as well as insights into the underlying mechanisms of AUD, which may be of significant implications for the exploitation of diagnostic biomarkers and gene therapy agents for AUD. The content outline of our present review was shown in **Figure 1**.

## Overview of ncRNAs

Thousands of unique ncRNAs sequences exist within cells, which constitute a sizeable portion of the transcriptional landscape 20, 21. According to the differences in size, structure, subcellular localization and biological properties, ncRNAs are generally categorized as regulatory ncRNAs and housekeeping ncRNAs 22. More specifically, housekeeping ncRNAs are the most fundamental and abundant RNAs that play important roles in the genomic transcription and translation, including transfer RNAs (tRNAs), ribosomal RNAs (rRNAs), small nucleolar RNAs (snoRNAs), small nuclear RNAs (snRNAs), etc., whereas regulatory ncRNAs are the functional components of gene expression with heterogeneous molecular groups, which can be further divided into microRNAs (miRNAs), small interfering RNAs (siRNAs), piwi interacting RNAs (piRNAs), long non coding RNAs (lncRNAs), circular RNAs (circRNAs) and natural antisense transcripts 23. These ncRNAs have long been regarded as transcriptional “junks RNA” when their expression, mechanism and function are unknown 24. In recent years, with the continuous development of emerging biotechnology and in-depth research on ncRNAs, scientific researchers have increasingly understood that ncRNAs are engaged in a variety of developmental and pathological associated cellular processes and pathways in vivo. Insights into the potential roles of ncRNAs in development and diseases, particularly in brain diseases, have made ncRNAs prospective tools and targets for novel therapeutic approaches. Dysregulation of these ncRNAs, especially lncRNAs, circRNAs and miRNAs, was consistently observed in AUD individuals, which may be associated with the onset and progression of AUD.

## CircRNAs and alcohol use disorder

CircRNAs are a kind of ncRNA with covalently closed loops and no polyadenylate tail and cap structure, allowing them to resist exonuclease degradation and so have a prolonged half-life 25. CircRNAs, originally discovered in pathogens, were subsequently found to be ubiquitously expressed in eukaryotes and have been closely linked to a variety of cellular and physiological processes through multiple mechanisms of actions, including competing endogenous RNAs (ceRNAs) network and alternative splicing 26. Although the majority of circRNAs were expressed at modest levels in most tissues, individual circRNAs can gradually accumulate to high levels in certain cell types and body fluids, particularly in brain and blood 27-29. Inspired by the characteristics of high abundance and evolutionary conservation, a growing body of research has highlighted the intriguing prospects of circRNAs as diagnostic markers and therapeutic targets for AUD.

**Figure 2:**
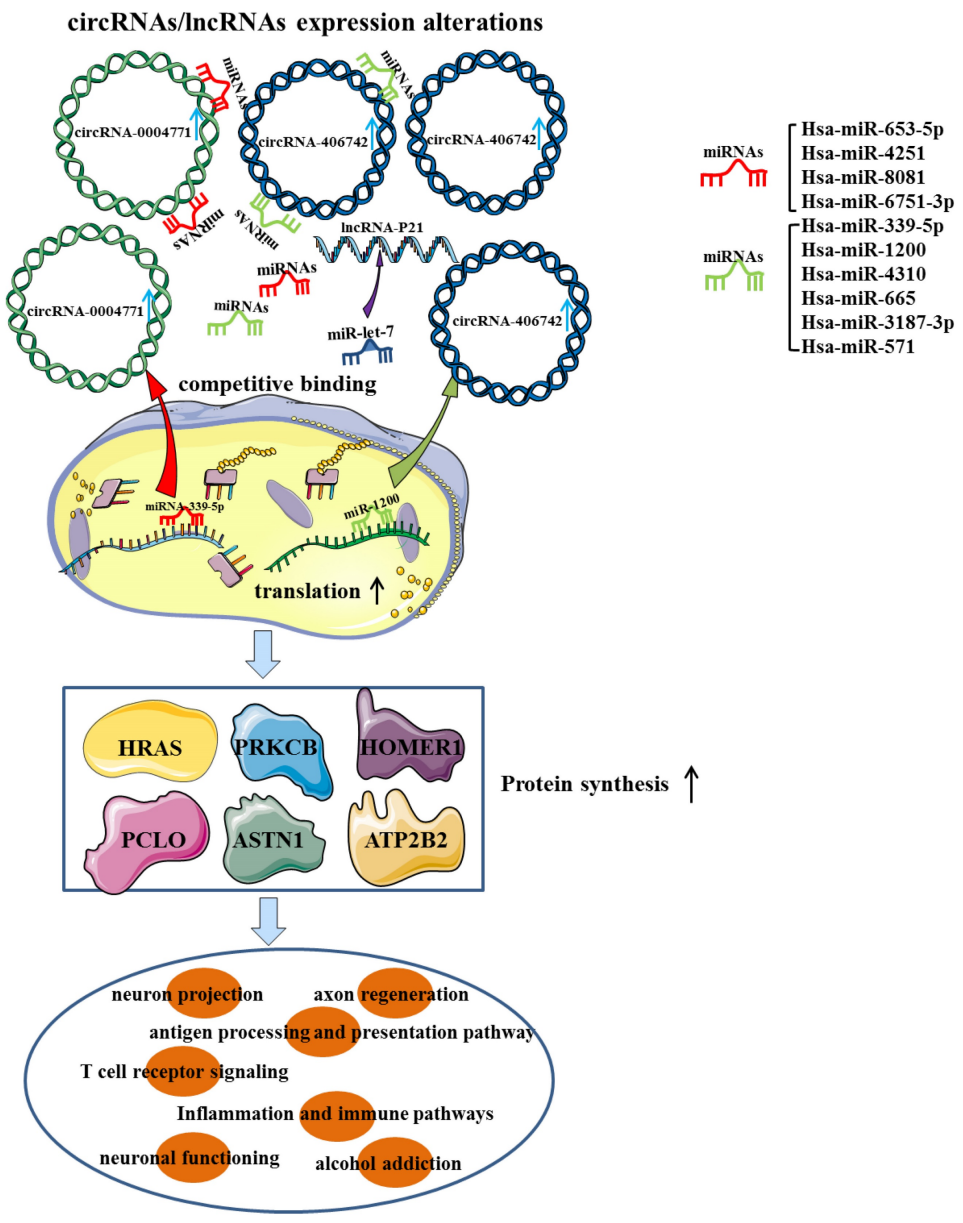
The potential ceRNAs (circRNAs/lncRNAs-miRNAs-mRNAs) network may be involved in the pathogenesis of alcohol use disorder.

High-throughput sequencing (HTS) identified a total of 399 improperly expressed circRNAs in brain samples from mice exposed to chronic intermittent ethanol (CIE), including 150 up-regulated circRNAs and 249 down-regulated circRNAs. Furthermore, bioinformatic analysis of circRNAs has demonstrated that these dyregulated circRNAs were mainly enriched in GABAergic synapse, retrograde endocannabinoid signaling and morphine addiction 30. CircRNAs within the exosomes are not easily degraded and can pass through the blood-brain barrier, making circulating exosomal circRNAs abundant and also of great research value in brain diseases 31. CircRNA-sequencing and quantitative real time PCR (qRT-PCR) results suggested that the expression of serum exosomal hsa-circ-0002130, 0000896, 0004771, 0000825 and 0007177 in AUD patients were significantly higher than those in healthy controls. Among them, hsa-circ-0004771 may be a sensitive diagnostic biomarker that was related to the severity of AUD and can effectively distinguish AUD patients from controls, with an area under curve (AUC) value of 0.874. Mechanistically, differentially expressed (DE) circRNAs may interact with several AD-related miRNAs and then affect infectious and inflammatory pathways 32. In another study of genome-wide expression of circRNAs in the nucleus accumbens (NAc) of 32 AUD cases/controls, circRNA-406742 expression was found to be decreased in AUD patients and negatively correlated with miR-1200 expression. The ceRNAs networks (circRNA-406742/ miR-1200/HRAS, HOMER1, PRKCB and PCLO) were thought to participate in the pathogenesis of AUD **(Figure 2)**. Besides, the cis-eQTLs of circRNA-000480, 001675, 104942 and 406742 were highly enriched in GWAS & Sequencing Consortium of Alcohol and Nicotine Use and Psychiatric Genetics Consortium AUD GWAS 33, 34. In conclusion, circRNAs functioned as miRNAs sponge to affect synaptic transmission and neural function through the regulation of target genes, providing a fundamental basis for future studies on the underlying mechanisms of AUD. **(Table 1)**.

## LncRNAs and alcohol use disorders

LncRNAs are one of the most abundant classes of ncRNAs in the brain with a length of more than 200 nucleotides and no open reading frames 35. It is well acknowledged that lncRNAs are of great biological significance in brain function and dysfunction, and that most of which are expressed in a tissue- and temporal- specific manner in the CNS 36. Evidence has increasingly accumulated that lncRNAs are not only dysregulated in a wide range of neurological illnesses, but also play regulatory roles in gene expression, cellular functions and pathological processes 37, 38. With the extensive use of modern biotechnologies and bioinformatics, dysregulated lncRNAs were frequently found in the brain of individuals with AUD and have also been related to the pathophysiology of AUD.

### The lncRNAs expression and their-related mechanisms in AUD individuals

LncRNA MALAT-1 was shown to be dramatically up-regulated in the cerebellum, hippocampus and brain stem of AUD individuals. Similarly, substantial upregulation of MALAT-1 was observed in the hippocampus and brain stem, as well as the cortex of AUD rats following alcohol withdrawal 39. There existed a positive correlation between MALAT-1 and neuropeptide 1, and both of them were noticeably enhanced in excitatory synapses and participated in the brain's reward response, which may increase alcohol craving and initiate AUD progression by forming complexes with PSD-95 and NMDA NR2 receptors 40. It was found that the stability of lncRNA-P21 was decreased in AUD rat model. The possible mechanism was that lncRNA P21 bound to the RNA-binding protein HuR and recruited let-7/AGO2, thereby targeting mRNA translation and boosting AUD development 40, 41 **(Figure 2)**.

**Figure 3:**
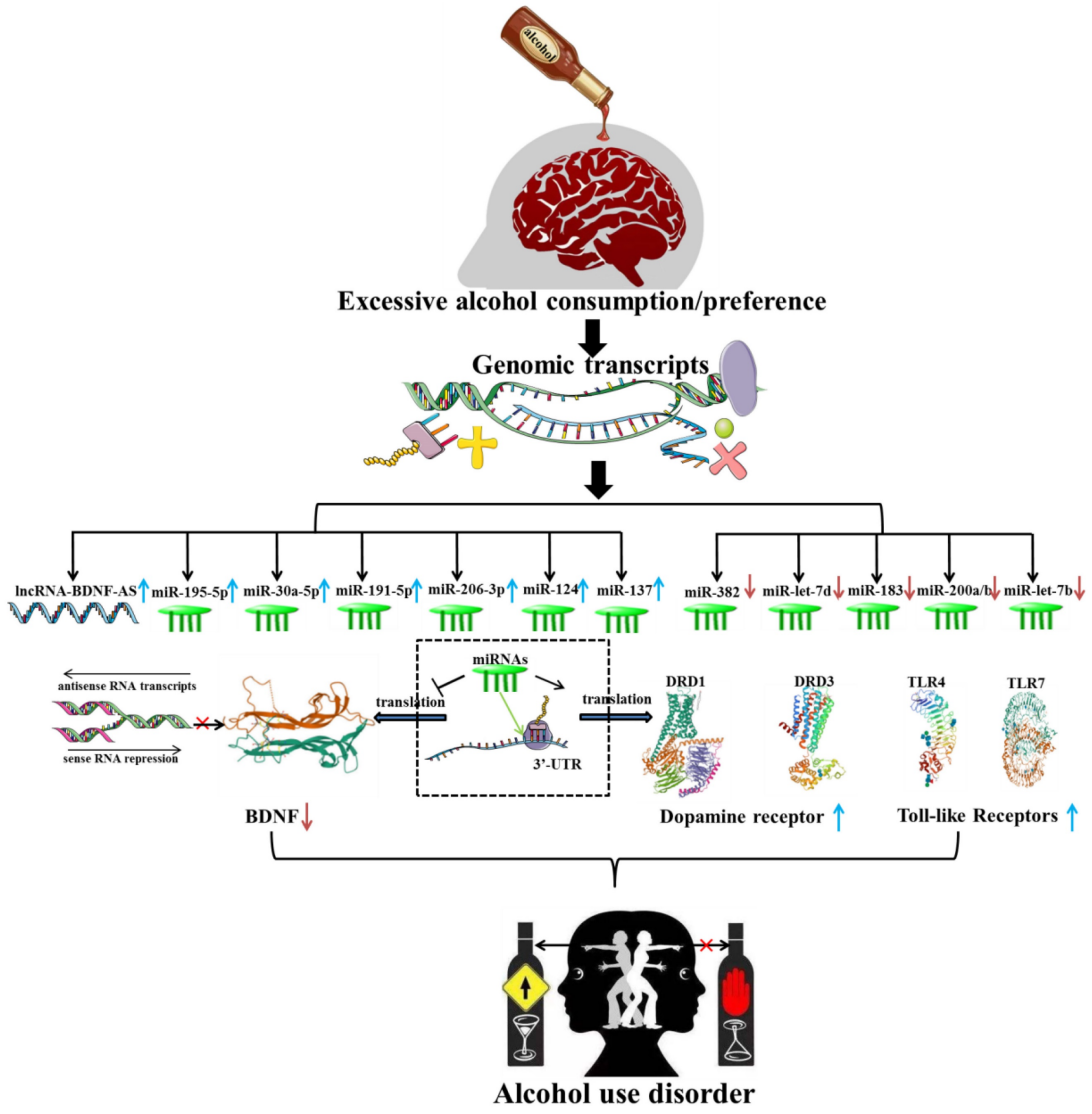
Several candidate miRNAs specifically target Brain-derived neurotrophic factor (BDNF), Dopamine receptor (DR) and Toll-like receptor (TLR), which are widely acknowledged to be associated with the occurrence and development of alcohol use disorder.

In recent years, several investigations have verified the aberrant expression of brain-derived neurotrophic growth factor (BDNF) in AUD patients and animal models 42, 43. LncRNA BDNF-AS, a naturally occurring BDNF antisense, was robustly elevated in early onset AUD amygdala (AMY), which appeared to account for decreased BDNF expression and increased EZH2 recruitment. Bohnsack *et al*. emphasized the importance of enhanced lncRNA BDNF-AS expression for synaptic plasticity and epigenetic reprogramming in the human AMY region 44 **(Figure 3)**. A recent transcriptome analysis has revealed the aberrant expression of lncRNAs, including lincRNA (SNORD3C, RP11-403B2.6, SNORD3C, RP11-638F5.1, RP4-723E3.1, SNORD3C), pseudogene (RP11-252O2.2, HSPA7, RP5-1033K19.2, KRT16P2, RP11-299H22.5) and antisense (RP13-126C7.1, RP11-543H23.2, RP5-1185I7.1) in different brain regions of AUD patients. Further mechanistic studies have shown that these lncRNAs were related to splicing factors dysfunction and may be involved in the course of AUD by regulating post-transcriptional processes and mature mRNA production 45. In an original study, Farri *et al.* indicated that several lncRNAs, including NCRNA-00092, 00174 and 00284, may have a cooperative role in cellular plasticity, ultimately leading to the formation of a fixed pattern of chronic alcohol abuse 46. Furthermore, RNA-seq has demonstrated that eleven lncRNAs (NCRNA-00051, 00256A, 00250A, 00256B, 00245, 00176, 00116, 00175, 00173, 00230B, 00107) were expressed substantially higher in the prefrontal cortex (PFC) of AUD individuals than in controls. While the expression of three lncRNAs (NCRNA-00169, 00161 and 00290) were significantly decreased in AUD individuals 47. Drake *et al*. found that 36 lncRNAs modules were obviously associated with AUD and were mostly abundant in alcohol-related processes such as microglia, postsynaptic membrane, glutamatergic synapse, basal neuron and immune-related systems 48. Saba *et al.* first revealed an unannotated transcript resembling a lncRNA, which was then termed as “Long noncoding RNA for alcohol preference” (Lrap). Genetic studies revealed that the disrupting Lrap may result in an escalation of alcohol consumption and a pronounced preference for alcohol through controlling gene expression and splicing of a large number of brain transcripts 49. Given the overlapping changes seen in different types of substance dependence 50, NEAT1, MIAT, MEG3 and EMX2OS, which were previously identified to be up-regulated in heroin/cocaine individuals, were proposed to be involved in the process of developing alcohol addition by modulating synaptic function, synaptogenesis and neuroadaptative mechanisms 51, 52. In conclusion, all these findings highlighted the potentially regulatory functions of lncRNAs in AUD.

Although numerous studies have identified brain transcriptomic patterns in AUD patients and animal models, the expression levels of transcripts in unique cell types have been rarely investigated. A recent scRNA-seq revealed that lncRNA FP700111.1, AC079793.1, AL807742.1, AC008957.2, AL022068.1, AL512598.2, AC098588.2, AC023421.2 AC104123.1, TMEM72-AS1, LINC01006 was elevated in GABAergic neurons, excitatory neurons, astrocytes, oligodendrocyte progenitor cells (OPCs), oligodendrocytes and microglia of AUD individuals, respectively. Ingenuity pathway analysis (IPA) showed that the identified DElncRNAs were predominately associated with neuroinflammatory signaling, GNRH signaling and neuroimmune response 53. **(Table 2)**.

### The lncRNAs risk locus associated with AUD

**Figure 4:**
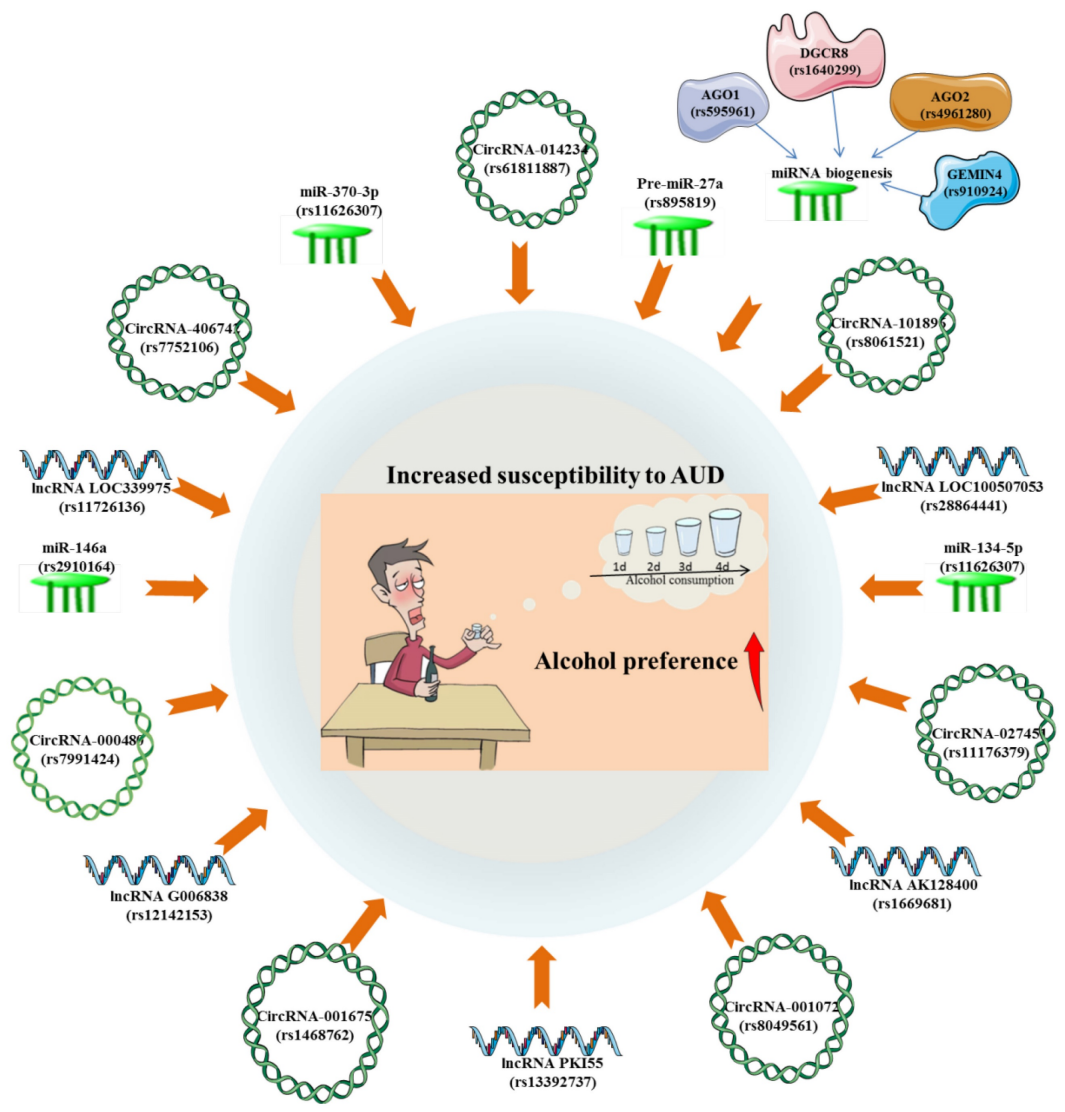
The significant associations between alcohol use disorder (susceptibility, consumption and preference) and SNPs of circRNAs, lncRNAs and miRNAs.

The GWAS of AUD has revealed the possible biological functions of the risk variants of related lncRNAs and their potential regulatory roles on gene expression. A prior GWAS observed an evident connection between lncRNA LOC100507053 locus (rs28864441) and AUD in African-Americans. Mechanistically, LOC100507053, as part of the larger ADH gene cluster, has the capacity to control numerous ADH genes through antisense function 54. Surprisingly, LOC339975 is classified as an enhancer lncRNA on chromosome 4 and may contribute to the increased ability of the chromosome to translate ADH 55. It was found that the associated allele of rs11726136 may be responsible for the decreased lncRNA LOC339975 expression in NAc, thereby participating in the pathogenesis of AUD through functional consequence 56. The cis-eQTLs analysis has demonstrated that the alternative allele of rs1669681 confers a lower lncRNA AK128400 expression and rs12142153 confers a higher lncRNA G006838 expression in AUD individuals, which might be important for elucidating the putative etiological mechanisms of AUD 48. Besides, there is experimental evidence that eQTLs for a lncRNA PKI55 locus (rs13392737) in the NAc were significantly enriched in AUD GWAS signals 57. **(Figure 4)**.

## MiRNAs and alcohol use disorder

MiRNAs are short endogenous single-stranded RNA molecules of 16-25 nucleotides that comprehensively regulate a variety of physiological and pathological processes in eukaryotic organisms 58. In terms of mechanisms of action, miRNAs primarily function in posttranscriptional regulation of target genes by inhibiting messenger RNA (mRNA) translation or promoting mRNA degradation 59. MiRNAs, highly abundant in the brain, are involved in multiple biological functions such as cell proliferation, differentiation, neurodegeneration, programmed death, DNA repair, self-renewal, synapse formation and plasticity, and show great potential as diagnostic markers and therapeutic targets for brain diseases 60. Over the past decades, genomics and genetics studies have identified a number of genes that may influence drinking behavior in humans and animal models. Notably, dysregulation of miRNAs lead to alterations in biological functions and cellular mechanisms, which may further contribute to the neuropathogenesis of AUD.

### The miRNAs expression and their-related mechanisms in AUD patients

The miRNAs expression profile and functional analysis revealed that several miRNAs were aberrantly expressed and involved in the progression of AUD through a variety of biological mechanisms. Lewohl *et al*. reported that about 35 miRNAs (hsa-miR-553/369-3p/18a/339-5p/1/7/196a/301a/144/let-7g/153/let-7f/203/34c-5p/101/376c/665/152/194/423-5p/515-3p/374b/140/519b-3p/586/135b/92a/15b/580/146a/454-3p/380/652/802/196b) were significantly up-regulated in the PFC of AUD individuals when compared with controls. It was then revealed that the predicted target genes of these up-regulated miRNAs have a great overlap with the mRNAs expression profile, thereby leading to the neuronal plasticity, deterioration and concomitant adaptation of neuronal functions 61. Besides, an original research emphasized that the expression of miR-34a and -34c in hippocampal postmortem tissue of AUD subjects were remarkably higher than those of controls 62. MiRNA expression profiling conducted by the Affymetrix GeneChip miRNA 2.0 revealed that 12 miRNAs (miR-493/29b/377/375/516a-2/299-3p/488/379/149/767-5p/105/3065-5p) were significantly up-regulated and 4 miRNAs (miR-3162/572/1227/2355) were down-regulated in the PFC of AUD subjects. Among them, 4 up-regulated miRNAs was located on chromosome 14q32, whose predicted gene targets are mainly involved with oligodendrocyte growth, differentiation and signaling 63.

The development of reliable, minimally invasive biomarkers in readily available body fluids may be beneficial for early prediction and diagnosis of advanced diseases. Small RNA-seq analysis identified that 7 miRNAs (miR-451a, miR-10a-5p, miR-100-5p, miR-3613-5p, miR-7704, miR-1290 and miR-4488) and 5 miRNAs (miR-126-3p, miR-10a-5p, miR-1290, miR-4488 and miR-1273h-5p) were differently expressed in the saliva of 56 African-Americans (AA) participants (28 AUD patients and 28 controls) and 64 European-Americans (EA) participants (32 AUD patients and 32 controls), respectively. These saliva miRNAs may be involved in DNA binding, alternative splicing or calcium-dependent cell-cell adhesion, and can serve as easily accessible biomarkers for prediction of AUD with the accuracy of 79.1% in AAs and 72.2% in EAs 64. Plasma miRNAs profiling revealed that the plasma concentrations of miR-122-5p, miR-3937, miR-193b-3p and miR-4507 were significantly associated with alcohol consumption and may play a mediatory role in the pathogenesis of AUD 65. Next generation sequencing identified that several serum miRNAs (miR-92b, miR-96, miR-24 and miR-136) were abnormally expressed in AUD individuals, which appeared to be synchronized with the changes in brain miRNAs expression. Ignacio et al. further suggested that these biomarkers may be responsible for alterations in CNS structure and function by consistently affecting cell death, cell proliferation and cell cycle processes 66. The extracellular release of miRNAs has been found to be one of the mechanisms by which ethanol modulates immunological activation, highlighting the important role of miRNAs in the pathogenesis of AUD. A prior study reported that miR-let-7b released from microglia-derived microvesicles may be responsible for microglial activation and increased Toll-like receptor 7 (TLR7) expression, which further contribute to the ethanol-induced neurotoxicity 67 **(Figure 3)**. MiRNA-Seq determined a number of upregulated circulating extracellular vesicle-bound miRNAs (EV-miRNAs) (miR-155/154/34c/450a/204/124a/211/96) and downregulated (EV-miRNAs) (miR-1224/625) as candidate biomarkers for chronic heavy drinking (CHD), whose expressions were found to be significantly associated with alcohol dose. Additional functional studies suggested that overexpression of miR-155, miR-154, miR-34c, miR-450a, and miR-204 could result in a heightened inflammatory response, while overexpression of miR-625 could lead to increased production of the inflammatory cytokines TNFα or IL-6 in CHD peripheral blood mononuclear cells (PBMC) followed by stimulation with PMA/ionomycin 68. The stress system has long been considered to play an essential role in motivating compulsive drinking, continued alcohol use and relapse. More recently, it has been revealed that up-regulation of psychological stress-related miR-10a, miR-21 and their downstream genes (transactivation responsive (TAR)-RNA-binding protein associated complex) may lead to increased alcohol intake in binge and alcohol abuse 69.

A large number of studies highlighted the critical roles of miRNAs-dependent repression of target genes and miRNAs-mRNAs regulatory networks in the pathogenesis of AUD. Small RNA-seq analysis determined 19 mature DEmiRNAs (miR-10a-5p, miR-182-5p, miR-1246, miR-126b-5p, miR-4485-3p, miR-486-3p, miR-486-5p, miR-144-5p, miR-1248, miR-5100, miR-1231, miR-144-3p, miR-122-5p, miR-412-5p, miR-4326, miR-302a-5p, miR-6868-3p, miR-5196-3p and miR-412-5p) and 97 DEmRNAs in reward-related or alcohol-responsive brain regions of AUD subjects and controls. It was then revealed that the miRNAs-mRNAs regulatory networks constructed using differential expression and negative correlation pairs could regulate AUD-related pathways, including CREB signaling, IL-8 signaling and axon guidance signaling, which might contribute to the onset and progression of AUD 70. A transcriptome analysis by microarray-based assay revealed the differential expression of miR-130a in postmortem PFC of 23 AUD subjects and 23 matched controls. Further functional annotation clustering analysis showed that the hub target genes (ITPR2 and ATP1A2) of miR-130a were responsible for the control of ion channel function, which may be involved in the etiological process of AUD 71. Although the miRNAs alterations in AUD patients showed great consistency, some controversies still existed. A recent meta-analysis of 6 clinical studies revealed that most miRNAs related to dopamine and gamma-aminobutyric acid receptor subunit were significantly up-regulated in AUD patients, while approximately 9% of miRNAs were down-regulated, including miR-567, miR-126, miR-1, miR-432, and miR-15 72. Undoubtedly, large-scale studies and more clinical work are needed if the screened specific miRNAs are to be clinically applied as potential biomarkers for AUD **(Table 3).**

### The miRNAs expression and their-related mechanisms in AUD animal models

Altered genes expression in the brains of alcohol addicts underlies the neural adaptations for alcoholism and compulsive drinking. The next-generation sequencing identified 14 known alcohol-sensitive miRNAs and 13 putative novel miRNAs in Drosophila repeatedly exposed to alcohol. Specifically, the overexpression of miR-6 and miR-310 were correlated with the increased ethanol sensitivity 73. Ethanol exposure may lead to upregulation of miR-153a/725/30d/let-7k/100/738/732 in Zebrafish embryos, which may further account for ethanol toxicology. Of course, whether alcohol exposure of Zebrafish embryos can be applied as an AUD model remains to be further validated 74. miR-7, miR-153, miR-152, miR-15B, miR-203 and miR-144 were predicted to target key genes implicated in the pathophysiology of AUD. The alterations in the expression of these mentioned miRNAs in different cell lines (HEK293T, SH SY5Y and 1321 N1 cells) following exposure to alcohol were consistent with those identified in postmortem brain of AUD patients. Therefore, Steenwyk et al. proposed that cellular models of AUD can be applied to illustrate the underlying mechanisms of gene expression changes that occurred in AUD patients, and that the exploration of effector cells and intercellular cascade mechanism for AUD is indispensable 75.

An original study supported that the expression of miR-411, miR-203, miR-137 and miR-92a were obviously reduced in the mPFC of AUD mice 76. It was widely acknowledged that the calcium- and voltage-gated BK channel was significantly associated with alcohol tolerance, which may lead to the development of AUD 77. Pietrzykowski *et al.* reported that chronic alcohol exposure lead to an increased expression of miR-9, which may participate in the alternative splicing and neuronal adaptation by down-regulating the expression of its target gene, the alpha subunit of the BK channel 78. miR-382, a critical novel gene, was found to be significantly down-regulated in both alcohol-treated neuronal cells and the NAc of AUD rats, and played a regulatory role in alcohol preference and voluntary intake through the negative regulation of the dopamine D1 receptor (DRD1) and DeltaFosB pathway 79. A recent research also reported that the expression of miR-382 was significantly reduced in the PFC area, which accompanied with an increase in DeltaFosB expression 80. The miR-lethal-7 (let-7) family are widely expressed in neurons throughout the brain and has been implicated in several neurophysiological functions and neurobiological responses to drugs abuse 81-83. Dreyer *et al*. previously found that miR-let-7d had a positive effect on reducing cocaine-induced conditioned place preference (CPP) and improving measures of anxiety- and depression-like behaviors in mice 84. Consistently, it was found that miR-let-7d expression was robustly reduced in AUD rats and inversely correlated with the alcohol intake and preference. Bahi *et al.* also hypothesized that the down-regulation of miR-let-7d might result in the elevated dopamine D3 receptor expression, which in turn enhance alcohol-related behavior responses 85 **(Figure 3)**.

It was generally accepted that the expression of BDNF was decreased in most neuropsychiatric disorders and negatively correlated with the severity of the diseases. Ehinger *et al.* indicated an inverse correlation between blood serum BDNF levels and alcohol intake in rapid-onset and late-onset AUD rats. In contrast, the expression of miR-30a-5p, miR-195-5p, miR-191-5p and miR-206-3p, which were known to target BDNF, were robustly elevated in the serum of rapid-onset AUD rats 86. It was found that the expression of miR-206 was significantly up-regulated in medial prefrontal cortex (mPFC) of rats with a history of AUD. Tapocik *et al.* also speculated that long-lasting induction of miR-206 expression may alter the neural circuits functions and synaptic plasticity through indirectly targeting BDNF 3'-UTR, which may contribute to escalated alcohol consumption 87. Another research article demonstrated that the expression of miR-206 was robustly increased, while the BDNF expression was reduced in mPFC, CeA and hippocampus of mice exposed to repeated cycles of CIE 88. It was also reported that the expression of miR-30a-5p in the mPFC of mice with excessive alcohol consumption was robustly up-regulated, which was accompanied by a decrease in BDNF expression. Moreover, the overexpression of miR-30a-5p conducted by a stereotaxic infusion of Ad-miR-30a-5p in the mPFC resulted in an escalation of alcohol intake and a preference for water in the two-bottle choice model, which may be attributed to the decreased BDNF expression 89. miR-124, which has also been reported to target BDNF, was found to be remarkably decreased after alcohol withdrawal in AUD mice, accompanied by a significant increase in the target gene CDC42, which may be responsible for the drinking relapse 90. A previous study reported that miR-124a was downregulated, while BDNF was upregulated in the dorsolateral striatum (DLS) of rats following voluntary alcohol intake. Surprisingly, exogenous overexpression of miR-124a mediated by lentiviral (LV)-miR-124a enhanced about fivefold ethamol-induced CPP and alcohol consumption, which may be induced by decreased BDNF expression 91. Ureña-Peralta *et al.* observed a significant down-regulation of miR-183 Cluster (miR-96/-182/-183) and miR-200a/b expression and an up-regulation of miR-125b in long-term chronic alcohol exposure WT mice. These miRNAs may lead to increased alcohol intake by regulating the voltage-gated sodium channel, neuron hyperexcitability, innate immune TLR4 signaling pathway, which eventually cause alcohol abuse 92 **(Figure 3)**.

Adolescence is a vulnerable period of neurodevelopment, and besides, alcohol exposure during pubertal development can lead to long-term and widespread neurobiological dysfunction, which may further be involved in the development of AUD. Binge drinking in adolescent is a potential risk factor for AUD and comorbid anxiety in adulthood. Kyzar *et al.* found that miR-137 was significantly upregulated and its target genes Lsd1 and Lsd1+8a were downregulated in the AMY of adolescent intermittent ethanol (AIE) adult, which may be caused by epigenetic reprogramming. As a result, they highlighted that miR-137 may be applied as a prospective predictive biomarker and therapeutic target for AUD-associated anxiety and AUD susceptibility in adulthood following adolescent alcohol exposure 93. Prins et al. indicated that peripubertal binge alcohol exposure has long term effects on the miRNAs (miR-10a-5p/26a/103/495) expression and further account for changes in hippocampal function, which may be mediated by the regulation of BDNF and sirtuin-1 (SIRT1) expression 94. MiRNAs within the EVs can cross the blood-brain barrier and are very stable in the peripheral circulation, which are widely used as reliable biomarkers for brain diseases. It has been confirmed that long-term ethanol exposure reduced the levels of miR-146a-5p and miR-21-5p, thereby triggering the high expression of inflammatory target genes (Traf6, Stat3 and Camk2a). These findings also supported the notion that circulating miRNAs may be involved in the early pathological process of adult AUD patients, such as ethanol-induced neuroinflammation and brain injury in adolescents 95. It has also been demonstrated that 6 miRNAs (miR-19a-3p/19b-3p/29a-3p/29c-3p/34a/488-3p) were significantly altered in rats exposed to repeated binge alcohol compared to control-treated counterparts 96.

Based on the emerging biotechnology and bioinformatics approaches, a great deal of studies revealed the coordinated dysregulation of miRNAs and associated mRNAs network in the AUD rats brain, which may be of great biological implications for the course of AUD. miRNA microarrays analysis showed that 41 rat miRNAs were significantly altered in the mPFC of rats after a history of AUD. Bioinformatics analysis further identified 33 miRNAs putatively targeting 89 mRNAs, which in turn affect neurotransmission, neuroadaptation, synaptic plasticity, axonal guidance and neurotransmitter signaling 97. Besides, integration analysis of ex vivo miRNAs and protein expression by Gorini *et al.* revealed that miRNAs (miR-488-3p/410-3p/3084-3p/let-7a-2-3p/200a-3p/140-3p/96-5p/3107-3p/34b-5p/141-3p/410-3p) were predominantly upregulated and mRNAs were downregulated in CIE-exposed dependent mice. Functional analysis suggested that the coordinated, synergistic regulation of miRNAs and proteins may be responsible for multiple neuroadaptations, driving the behavioral transition from alcohol consumption to dependence 98. Nunez et al. first reported a positive correlation between increased miRNAs expression and increased mRNA expression in the early stage of AUD, shedding novel light on the current understanding of AUD pathogenesis. They also speculated that the early activation of miRNAs (let-7, miR-7, miR-15, miR-101, miR-140, miR-152, miR-17, miR-34, miR-135, miR-144, miR-146, miR-301, miR-339 and miR-368) may be an adaptive regulation in the initial stage of AUD 99. It was also demonstrated that long-term ethanol exposure can lead to temporal- and brain area-dependent dysregulation of complex gene networks in C57BL/6J mice. Surprisingly, three DEmiRNAs (miR-187-3p, miR-2137 and miR-7b-3p) were generally detected in AMY, NAc and PFC regions 100. The expression profiles revealed 11 miRNAs (miR-182, miR-382, miR-9, miR-543, miR-483, miR-206, miR-130a, miR-16, miR-30a and miR27a) and 9 mRNAs (BDNF, SATB2, NRD4A2, GALR1, CREB, SLC17A7-8, ARC) whose expressions were significantly altered after alcohol dependency and withdrawal in rats. Integrated variations of miRNAs-mRNAs expressions in rat brain were linked to pathways, such as reward pathways, synaptic plasticity, neuron differentiation, neurotransmission and chromatin organization, thereby reconstituting the neural circuit function required for the development of AUD 101. A genome-wide analysis of miRNAs expression identified that AUD-associated miRNAs modules showed significant enrichment for both neuronal, astrocyte and glial marker genes. Among them, miR-449a/b were found to have additional functions related to cellular proliferation and neurotoxic effects in the brain, and may be involved in the pathogenesis of AUD through the negative regulation of hub genes (ELAVL4, DPYSL3, and KCNJ6) in the NAc 102. A recent study by Martinez reported that the expressions of miR-9-3p, 15b-5p, 16-5p and 222-3p were upregulated in the ethanol-drinking rats model and were almost unchanged in the groups with simultaneous ingestion of ethanol and caffeine. These findings only appeared to indicate that greater caffeine consumption in rats may result in weak changes in serum miRNAs that do not counteract the genetic changes caused by AUD 81 **(Table 4).**

### The miRNAs risk locus associated with AUD

More and more studies have emphasized that there is a close relationship between genotype polymorphism and phenotypic diversity, all of which are of significant importance for understanding the pathogenesis of diseases and predicting the outcome of diseases. A genetic association study described that individuals who are allele C carriers of the miR-146a G>C polymorphism (rs2910164) were susceptible to AUD 103. Besides, it was found that the Pre-miR-27a rs895819 A>G polymorphism were significantly associated with heavy alcohol consumption in a Mediterranean population, which may account for the occurrence of AUD 104. Weighted gene co-expression network analyses (WGCNA) on miRNAs expression in NAc of AUD identified 3 significant miRNAs modules and 25 miRNAs hub genes, including hsa-miR-34b-5p/34c-5p/34c-3p/375/34b-3p/4652-3p/4423-3p/383-5p/212-3p/377-5p/132-3p/1912/1180-3p/382-5p/370-3p/361-5p/4760-3p/3189-5p/134-5p/523-3p/4720-3p/4633-5p/4762-5p/4311/555. It was then observed that the significant eQTLs for these 25 miRNA hubs were predominantly enriched in alcohol related GWAS 57. Among them, hsa-miR-34 family miRNAs have been shown to possibly exhibit trans-eQTL effects with variants associated with NNAT and PSMB5, which may be implicated in the pathogenesis of AUD 57, 105. There existed a significant association between genes variants of miRNA biogenesis and AUD risk. Specifically, it was found that AGO1 rs595961, AGO2 rs4961280 and DGCR8 rs1640299 have significantly altered the risk for AUD 106. These findings highlighted the potential relevance of polymorphisms within miRNAs genes as prospective biomarkers to illustrate the susceptibility to AUD **(Figure 4)**.

### The potential therapeutic effects of miRNA-relevant biological agents on AUD

Gene therapy is considered to be the third revolution in the field of medicine and pharmacy because it can fundamentally prevent, cure or alleviate various genetic diseases 107. Detecting gene mutations and comprehending the specific molecular mechanism of hereditary disorders, the type of genetic modifications and delivery methods are critical procedures to perform gene therapy. Gene therapy drugs, including DNA drugs based on DNA modification (e.g., in vivo gene therapy drugs based on viral vectors, in vitro gene therapy drugs, naked plasmid drugs, etc.) and RNA drugs (e.g., antisense oligonucleotide (ASO) drugs, RNA interference drugs, mRNA gene therapy, etc.), are promising therapeutic tools 108. After years of tortuous development, a total of about 43 gene therapy drugs have been approved for clinical application and more than 3,000 preclinical and clinical trials of gene therapy are under way or have been completed worldwide 109. Beyond that, in-vivo gene therapy based on viral vectors and small nucleic acid drugs (ASO, siRNA and miRNA) have made significant progresses in preclinical and clinical trials of AUD, which have greatly revolutionized the field and are garnering fresh interest.

**Figure 5:**
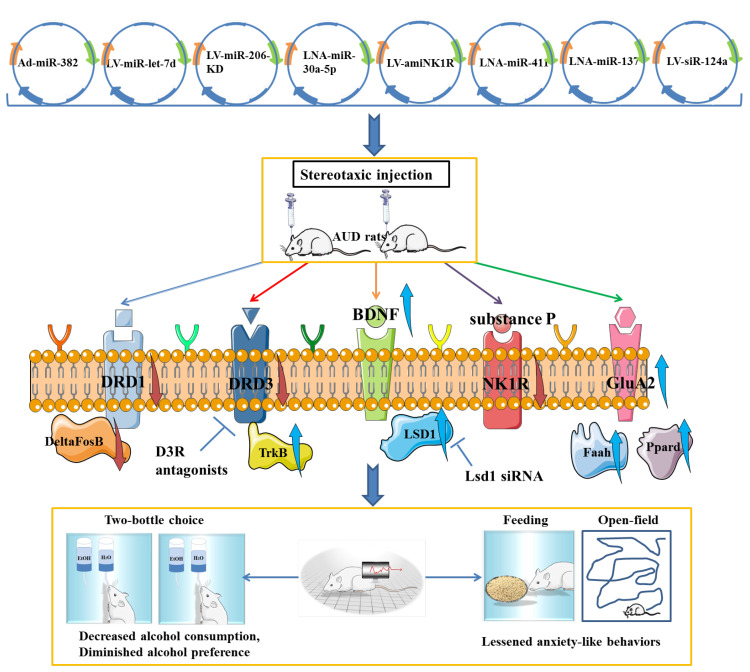
The positive effects of miRNAs-associated biotechnological agents on attenuating alcohol preference, decreasing voluntary alcohol intake and lessening anxiety-like behaviors, as well as the involvement of potential regulatory mechanisms mediated by the gene therapy agents in alcohol use disorder.

It was confirmed that overexpression of miR-382 via adenovirus (Ad)-miR-382 could effectively affect the drinking behavior and responses, such as lowering voluntary alcohol intake and the preference for alcohol in rats under the intermittent access two-bottle choice drinking paradigm 79. LV-mediated up-regulation of miR-let-7d in accumbal could effectively decrease alcohol preference and intake by directly targeting the dopamine D3 receptor to decrease its expression 85. There is experimental evidence that viral-mediated overexpression of miR-206 in the mPFC was sufficient to induce the escalation of alcohol self-administration of rats. Given the causal role of low expression of miR-206 in the induction of BDNF expression and recovery of neural function, it can be speculated that miR-206 could be a potential therapeutic target for AUD 87. Tapocik *et al.* have not indicated the potential effect of miRNA-206 knockdown on alcohol intake and alcohol preference in AUD rats, which probably due to the complexity of knockout techniques in vivo. Furthermore, Darcq *et al.* indicated that inhibition of miR-30a-5p in the mPFC using a locked nucleic acid sequence could decrease excessive alcohol intake and restore the BDNF expression, while overexpression of miR-30a-5p could produce an escalation of alcohol intake and a preference for alcohol 89. The neuropeptide substance P and neurokinin-1 receptor (NK1R) are widely acknowledged to be involved in the stress response and drug reward systems. The artificial miRNA (amiRNA) carried by lentiviral vector that target the NK1R effectively decreased the voluntary alcohol consumption of AUD rats and NK1R expression in the hippocampus, positively supporting the potential use of amiRNA as a therapeutic agent for the treatment of AUD 110. It was confirmed that inhibition of miR-137 through direct infusion of antagomiR-137 into the AMY could effectively rescue AIE-induced alcohol intake and anxiety-like behaviors. Further mechanistic studies showed that silencing of miR-137 has an effect on normalizing the decreased LSD1 expression, decreased LSD1 occupancy and decreased BDNF IV expression via increasing H3K9 dimethylation in AIE adult rats 93. Interestingly, knockdown of miR-124a expression via LV-siR-124a infusion in the DLS positively attenuate ethanol-induced CPP and voluntary alcohol consumption, which was similar to the regulatory effect of LV-BDNF infusion 91. Although miR-411 was found to be reduced in mice exposed to a chronic alcohol drinking paradigm, silencing of miR-411 by infusion of antagomiR-411 into the mPFC could decrease voluntary alcohol consumption and preference of mice with a history of AUD, which may be related to the elevated levels of glutamate receptor AMPA-2 subunit (GluA2), fatty acid amide hydrolase (Faah) and peroxisome proliferator activated receptor delta (Ppard) 76. In conclusion, all these findings positively supported the involvement of miRNAs in AUD pathogenesis and may represent novel therapeutic targets for AUD **(Figure 5).**

Taken together, gene therapy agents can effectively ameliorate AUD-related symptoms by mimicking or silencing AUD-related miRNAs, providing theoretical guidance and practical reference for exogenous regulation of miRNAs in the treatment of AUD. Once the effectiveness of the miRNAs-target agents is experimentally verified, their safety and delivery efficiency can be further optimized. Notably, multiple significant advancements in preclinical models are required to convert preclinical research into clinical practice. Firstly, the safety of gene transfection system, such as host immune response and long-term toxicity, deserved to be great concern. The second issue that needs to be addressed is the effectiveness of the objective genes. The current reality is that most gene therapies are non-specific. The stability and targeting of gene transmission systems are other important restrictions that prevent gene therapy techniques from prospering. In addition, the development and optimization of efficient, safe and targeted gene delivery vectors are also indispensable. The last and most critical constraint is the species difference between humans and model animals, which may result in dramatically different treatment outcomes. In the near future, gene therapy will eventually shine in the treatment of refractory diseases with the continuous development of emerging biotechnologies and materials.

## Conclusions and future perspective

Alcohol use disorder (AUD) is an intractable global public health issue with high morbidity, disability and mortality. The current situation of AUD diagnosis and treatment is alarming because of the low rate of consultation and treatment success. Genetic alterations and epigenetic modifications have been associated with the susceptibility to AUD and are involved in the pathogenesis of AUD. Concretely, γ-aminobutyric acid (GABA) transporter 3 (GAT-3) was confirmed to be selectively reduced in the central AMY of AUD patients, accompanied by the impaired GABA clearance 111. An elevated level of TLR4 signal was found in the ventral tegmental area (VTA) of alcohol-preferring (P) rats in a prior research 112. In addition, RNA methylation analysis by Arraystar has revealed 29 mRNAs, 5 lncRNAs, and 3 miRNAs that were differently methylated in postmortem NAc of AUD patients and controls 113. Moonat *et al.* emphasized the crucial roles of aberrant histone deacetylase 2-mediated histone modifications and synaptic plasticity within the AMY in the pathogenesis of AUD 114. It has been widely reported that biological processes such as oxidative stress, GABAergic neurotransmission and neuroimmune were strongly related to the onset and progression of AUD 115-117. Nevertheless, there is still a lack of research on the relationships between ncRNAs and genetic and epigenetic mechanisms, as well as the possible involvement of ncRNAs-mediated cellular functions and biological processes in the course of AUD. Most noteworthy, whether these biological mechanisms are governed by the upstream ncRNAs is another study avenue.

A large number of ncRNAs have been implicated in the onset and progression of drug addiction 118. Whether these ncRNAs can be used as diagnostic indicators and therapeutic targets for AUD are also worthy of further study. Given the recent finding of peptides and proteins encoded by ncRNAs in tumors 119, it is worth investigating whether peptides and proteins encoded by ncRNAs might potentially serve as prospective diagnostic biomarkers and pharmacological targets for AUD. Once validated, these findings will provide a wealth of opportunities for biomarkers, targets and gene therapy drugs. Exosomes participated in cell-to-cell transmission of information and substances through particular mechanisms, and have shown great application advantages and prospects in the diagnosis and treatment of various diseases 120. Owing to the good stability, biological activity, targeting specificity, and easy crossing of the blood-brain barrier, circulating exosomes are thought to be a valuable model for understanding the pathogenesis of brain disorders 121. In fact, studies on the potential roles and regulatory mechanisms of exosomal ncRNAs in AUD are particularly lacking, which may point to a weak avenue for understanding the pathophysiology of AUD. More recently, we have performed plasma exosomal miRNA-seq analysis on 25 clinical samples and identified a large number of differentially expressed circulating exosomal miRNAs that could effectively distinguish AUD from normal controls.

Advances in the field of gene therapy have raised the prospect of curing resistant diseases. As we reviewed, the favorable effects of miRNAs-associated gene therapy agents on alleviating alcohol preference and intake have suggested that miRNAs may be prospective therapeutic targets for AUD. However, it is worth evaluating whether therapeutic agents delivered by adenovirus, lentivirus vector and stereotaxic injection might cause substantial damage to rats and affect their alcohol self-administration. Exosomes, as favorable non-viral gene delivery carriers, can protect gene material from the host immune response, and has the advantages of high biocompatibility, low clearance rate, strong permeation retention effect, and cell-targeted delivery. In the future, engineered exosomes can be designed for tissue- and cell-specific delivery, such as specific recognition sequences installed outside exosomes or protein-protein interactions designed by exosomal proteins to enrich exosomes, thereby improving the targeting effect of exosomes as well as loading and delivery efficiency, which may represent a reliable strategy for AUD therapy. Encouragingly, exosomes engineering is gradually being applied preclinical studies, which has shown great clinical benefits and a broad applicability promise. It's also worth noting that neither an escalation nor an inhibition of alcohol intake and preference experiments were performed in transgenic mice. Furthermore, the current reality is that therapeutic effects have only been studied in animal models of AUD, and have not risen to the height of human trials, which deserved to be further investigated. To our knowledge, some other types of ncRNAs such as piRNAs, snRNAs and snoRNAs have been identified to be significantly associated with the progression of certain diseases, which may also play an unforeseen role in the pathogenesis of AUD.

Stereotactic injection may have higher selectivity and regulatory efficiency than systemic injection, which is conducive to safer and more efficient role of gene therapy agents in the treatment of AUD. The pathophysiological processes of brain diseases such as AUD may be precisely pinpointed to a certain brain region. It may lead to poor therapeutic efficiency and significant disparities in therapeutic outcomes among different species if gene therapy agents delivered by exosomes cannot precisely navigate to specific brain regions. But fortunately, systemic administration of EV-derived miR-124 has been shown to attenuate radiation-induced brain injury and secondary neuroinflammation 122. Another study has also demonstrated that systemic administration of rabies virus glycoprotein (RVG)-exosomes loaded with miR-124 effectively promoted cortical neurogenesis and protected against ischemic injury 123. In addition, it was found that systemic antimiR-337-3p delivery has a favorable rescue effect on cerebral ischemia-mediated damage 124. Lai* et al.* reported that systemic exosomal miR-193b-3p delivery dramatically alleviated neuroinflammation in early brain damage following subarachnoid hemorrhage in mice 125. Although some other studies have also shown that systemic delivery of miRNA-related gene therapy agents has a favorable effect on brain diseases, the existence of blood-brain barrier may have a slight impact on the therapeutic effect of systemic delivery. Due to its capacity to quickly cross the blood-brain barrier, exosomes delivery vectors can be carefully designed to localize to the induced region of AUD, thereby providing a unique therapeutic benefit.

Despite substantial efforts, only a few gene therapy agents have been approved for clinical use due to many challenges such as potential genotoxicity, inefficiencies in gene transfer or editing, immune responses to repeated drug delivery vectors, and a lack of a societal consensus on controversial issues. Over the past few years, the scientific advances and clinical successes have demonstrated the potential for lasting human health benefits gained from gene therapy, so we have good grounds to remain optimistic and step up efforts to make this therapy part of our standard approach to treating major human diseases. In conclusion, with the emergence and widespread application of modern biotechnology, such as next generation sequencing, microarray, scRNA-seq, GWAS and engineered exosomes, we will finally make breakthroughs in the investigation of the pathogenesis of AUD and the development of therapeutic drugs. Gene therapy could one day upend current treatment strategies for genetic diseases by the use of precise genetic manipulation and delivery tools.

## Figures and Tables

**Table 1:** CircRNAs in alcohol use disorder

CircRNAs	Source	Species	Expression	Target	Enriched pathways	References
circ_0002130	Serum, Exosomes	Human	Up	miR-6074/miR-6514-5p/miR-662/miR-326/miR-4482-3p		
circ_0000896	Serum, Exosomes	Human	Up	miR-4498/miR-3692-5p/miR-1270/miR-5001-5p/miR-4492		
circ_0004771	Serum, Exosomes	Human	Up	miR-8081/miR-6751-3p/ miR-339-5p/miR-4251/miR-653-5p		
circ_0000825	Serum, Exosomes	Human	Up	miR-340-3p/miR-30a-5p/ miR-4686/miR-4724-3p/miR-5195-3p	T cell receptor signaling, Antigen processing, presentation pathways	32
circ_0007177	Serum, Exosomes	Human	Up	miR-224-5p/miR-514a-5p/miR-6891-3p/miR-670-3p/miR-153-5p		
circ_002910	Serum, Exosomes	Human	Down	miR-1258/miR-6812-5p/miR-6751-5p/miR-6855-5p/miR-6803-5p		
circ_004794	Serum, Exosomes	Human	Down	miR-6782-5p/miR-6894-5p/miR-30b-3p/miR-6751-5p/miR-6796-5p		
circ_406742	Nucleus accumbens	Human	Down	miR-1200		
circ_000390	Nucleus accumbens	Human	Down	miR-361-5p		
circ_065645	Nucleus accumbens	Human	Down	miR-571	synaptic transmission, cellular localization,	33, 34
circ_405170	Nucleus accumbens	Human	Down	miR-4310	neural development	
circ_101134	Nucleus accumbens	Human	Down	miR-665		
circ_001072	Nucleus accumbens	Human	Down	miR-3187-3p		
150 circRNAs	Brain samples	Mice	Up	NM	morphine addiction GABAergic synapse	
249 circRNAs	Brain samples	Mice	Down	NM	Endocannabinoid signaling	30

NM: Not mentioned

**Table 2:** LncRNAs in alcohol use disorder

LncRNAs	Source	Species	Expression	Target	Pathways	References
MALAT-1	Cerebellum Hippocampus Brain stem	Human	Up	Neuropeptide 1	Excitatory synapses Brain reward response	39
P21	Brain tissue	Rats	Down	miR-let-7	Neuropeptides activation, Neurotoxicity	40, 41
BDNF-AS	Amygdala	Human	Up	BDNF/E2H2	Synaptic plasticity, Epigenetic reprogramming	44
SNORD3C/ RP11-403B2.6/ SH3RF3-AS1/ HSPA7	Superior frontal cortex	Human	Up	Specific splicing factors	Dysregulation of splicing factors	45
RP11-252O2.2	Nucleus accumbens	Human	Up			
RP11-543H23.2/ RP11-258F1.1/ TBL1XR1-AS1/ AFAP1-AS1/RP11-61I13.3/ AC137932.6/ SNORD3C/RP11-638F5.1/ FAM225B/ LINC00313/RP4-723E3.1/ HSPA7/ RPLP0P2/ RP5-1033K19.2/KRT16P2/ CTD-2575K13.6/AC000367.1/RP11-299H22.5	Basolateral amygdala	Human	Up			
RP11-543H23.2/ RP11-258F1.1/ RP5-1185I7.1/ RP11-350G8.5/RP13-126C7.1/ SNORD3C/ HSPA7/RPLP0P2/MTND6P6	Central nucleus of the amygdala	Human	Up			
NCRNA00092/NCRNA00174/NCRNA00284	Brain tissue	Human	NM	NM	Cellular plasticity	46
NCRNA-00051/00256A/00250A/00256B/00245/00176/00116/ 00175/00173/00230B/00107	Prefrontal cortex	Human	Up	NM	NM	47
NCRNA00169/NCRNA00161/NCRNA00290	Prefrontal cortex	Human	Down			
Lrap	Brain tissues	Rats	Down	P2rx4	Dysregulation of splicing factors	49
MEG3/MIAT/NEAT1/EMX2OS	Nucleus accumbens	Human	Up	NM	Synaptic function, Synaptogenesis, Neuroadaptative	51, 52
FP700111.1	GABAergic neurons	Human	Up	NM	Neuroinflammatory, GNRH signaling, Neuroimmune response	53
AC079793.1, AL807742.1	Excitatory neurons	Human	Up	NM		
AC008957.2, AL022068.1, AL512598.2, AC098588.2, AC023421.2, AC104123.1	Astrocytes	Human	Up	SLC1A3		
TMEM72-AS1, LINC01006	Oligodendrocytes	Human	Up	NM		
AP002453.1	Excitatory neurons	Human	Down	NM		
AC069228.1	Oligodendrocytes	Human	Down	NM		
AC011586.2	microglia	Human	Down	NM		

NM: Not mentioned.

**Table 3:** MiRNAs in primates with alcohol use disorder

MiRNAs	Source	Species	Expression	Target	Pathways	References
miR-553/369-3p/18a/339-5p/1/7/196a/301a/144/let-7g/153/let-7f/203/34c-5p/101/376c/665/152/194/423-5p/515-3p/374b/140/519b-3p/586/135b/92a/15b/580/146a/454-3p/380/652/802/196b	Prefrontal cortex	Human	Up	CXCR4/DICER1/BIRC6/SCARB2/EDIL3/SLAIN1/SSR1/GART/FAM108B1/RNF103/FBXL3/TEX2/FRYL/PAPD4/SESTD1/RIPK2/CDKN1B	Neuronal plasticity, Neuronal deterioration, Neuronal adaptation	61
miR-34a/miR-34c	Hippocampal tissue	Human	Up	NM	Neuronal physiology, pathology	62
miR-451a/10a-5p/100-5p/3613-5p/126-3p	Saliva	Human	Up	NM	DNA binding, Alternative splicing, Calcium-dependent cell-cell adhesion	64
miR-1290/1273h-5p/ 7704/4488	Saliva	Human	Down	NM	DNA binding, Alternative splicing, Calcium-dependent cell-cell adhesion	64
miR-96/320b-1/1976/24-1/30a/96-5p/127/136/320b-2/421/671/3615/3676	Serum	Human	Up	p53 and TNF	Cell proliferation, Cell cycle processes, CNS structure, function	66
miR-let-7b	Hippocampal tissue	Human	Up	TLR7	Neurotoxicity	67
miR-10a/ miR-21	Peripheral blood	Human	Up	TRBP	Autoimmune, HPA axis reactivity	69
miR-10a-5p/182-5p/1246/126b-5p/ 4485-3p/486-3p/486-5p/144-5p/124/5100/1231/144-3p/	Brain tissues	Human	Up	GPRC5C/PTH1R/PIM1/GPR75/CDKN1A/VEGFA/ADRB1/IFNLR1 THBS1/GPR88/JAK3/ITGA9/KCNJ12/VIPR1	CREB pathways, IL-8 pathways, Axon guidance, neuroplasticity	70
miR-122-5p/412-5p/4326/302a-5p/6868-3p/5196-3p/412-5p	Brain tissues	Human	Down	PLD1/FNBP1/CDH1/MMP15/TUBB6/GIPR/PDE8A/GPR27	Opioid pathways, G-Protein receptor	70
miR-193b-3p/122-5/3937	Plasma	Human	Up	ALDH2/ FLI/SMAD3/ XPO6/SLC7A11/ FOXP1	Transmembrane receptor protein Serine/Threonine kinase	65
miR-4507	Plasma	Human	Down	ADAM19/C16orf95	Cell surface receptor	65
miR-493/29b/377/375/516a-2/299-3p/488/379/149/767-5p/105/3065-5p	Frontal cortex	Human	Up	THBS2/CHN2/NDE1/UGT8/CNP/ENPP2/SEMA4D1	Oligodendrocyte growth, Differentiation, Neuronal migration	63
miR-3162/572/1227/2355	Frontal cortex	Human	Down			
miR-130a	Prefrontal cortex	Human	Down	ITPR2/ATP1A2/ JARID2	ion channel function, Neuroadaptations	71
miR-155/154/34c/450a/204/124a/211/96	Extracellular vesicle, Plasma	Macaque	Up	TNFα or IL-6	Inflammation, Immune activation	68]
miR-1224/625	Extracellular vesicle, Plasma	Macaque	Down			

NM: Not mentioned.

**Table 4:** MiRNAs in animals with alcohol use disorders

MiRNAs	Source	Species	Expression	Target	Pathways	References
miR-411/ 203/ 137/ 92a	Prefrontal cortex	Mice	Down	GluA2/Faah/Ppard	Neuroadaptations	76
miR-9	Brain tissue	Rat	Up	BK channel	Alternative splicing, Neuronal adaptation, Neuronal plasticity	78
miR-382	Nucleus accumbens	Rat	Down	DRD1	DeltaFosB pathways	79
miR-let-7d	Hippocampus	Mice	Down	DRD3	Epigenetic regulation, neuropathophysiology	85
miR-30a-5p/195-5p/ 191-5p/206-3p	Serum	Rat	Up	BDNF	Neuronal differentiation, Synapse formation, Synaptic plasticity	86
miR-206	Prefrontal cortex	Rat	Up	BDNF	Neuroadaptations	87
miR-30a-5p	Prefrontal cortex	Rat	Up	BDNF	Neuronal proliferation, differentiation, survival, Synaptic plasticity	89
miR-124	Brain tissue	Mouse	Down	Cdc42	Neuronal development	90
miR-488-3p/410-3p/3084-3p/let-7a-2-3p/200a-3p/140-3p/96-5p/3107-3p/34b-5p/141-3p/410-3p	Brain tissue	Mice	Up	DNM1L/HS90A/DNM1L/DPYL3/DYN1 and so on	Neuroadaptations	98
miR-let-7/7/15/101/140/152/17/34/135/144/146/301/339/368	Frontal cortex	Mouse	Up	Aak1/Alcam/Cdc42/Scd1/Mlf2/Gtdc1/Kifc2/Mat2b and so on	Adaptive response	99
miR-187-3p/2137/7b-3p	Brain tissue	Mice	Down	Mrpl49/Eml5/Bicd2/Acta2/Arhgap44/Dusp/Ccdc149/Fbxl8 Polr2d and so on	Neurotoxicity, Neuronal function	100
miR-182/382/9/543/483/206/130a/16/30a/27a	Brain tissue	Mice	Up/Down	BDNF/SATB2/NRD4A2/GALR1/CREB/SLC17A7-8/ARC	Reward pathways, Synaptic plasticity, Neurotransmission	101
miR-449a/b	Nucleus accumbens	Mice	Up	ELAVL4/DPYSL3/KCNJ6	Neurobiological processes	102
miR-9-3p/15b-5p/16-5p/222-3p	Serum	Rat	Up	Caspase-3/XIAP/IGF-1R	Neurogenesis, Neuroprotector effects	81
miR-96/182/183/200a/200b	Cerebral cortex	Mice	Down	Nav1.3/Trpv1/Smad3/PP1-γ/Il1r1/Mapk14/Sirt1/Lrp6/BDNF	Neuroinflammatory pathways	92
miR-125b	Cerebral cortex	Mice	Up			
miR-137	Amygdala	Rats	Up	LSD1/BDNF	Chromatin remodeling, H3K9 dimethylation	93
miR-124a	Dorsolateral striatum	Rats	Down	BDNF	TrkB signaling, DRD3 pathways	91
miR-10a-5p/26a/103/495	Hippocampus	Rats	Up	BDNF/SIRT1	Neuronal development, Neurogenesis	94
miR-146a-5p/ miR-21-5p	Extracellular vesicles, Plasma	Rats	Down	Traf6/Stat3/Camk2a	Neuroinflammation, Neurotoxicity	95
miR-19a-3p/19b-3p/29a-3p/29c-3p/34a/488-3p	Hippocampus	Rats	Down	ATXN1/KCNC3/VAMP2/VDAC1	Synaptic plasticity, Memory formation	96

## Abbreviations

AUD: alcohol use disorder
ncRNAs: noncoding RNAs
circRNAs: circular RNAs
lncRNAs: long noncoding RNAs
miRNAs: microRNAs
SNPs: single nucleotide polymorphisms
WE: Wernicke encephalopathy
DSM: Diagnostic and Statistical Manual of Mental Disorders
ICD: International Classification of Disease
GWAS: genome-wide association studies
DE: differently expressed
CIE: chronic intermittent ethanol
CNS: central nervous system
ceRNAs: competing endogenous RNAs
AUC: area under curve
WGCNA: weighted gene co-expression network analyses
HTS: High-throughput sequencing
NAc: nucleus accumbens
BDNF: brain-derived neurotrophic growth factor
AMY: amygdala
PFC: prefrontal cortex
scRNA-seq: single-cell RNA sequencing
DR: dopamine receptor
TLR: Toll-like receptor
NK1R: neurokinin-1 receptor
EV: extracellular vesicle
CHD: chronic heavy drinking
PBMC: peripheral blood mononuclear cells
amiRNA: artificial miRNA
GABA: γ-aminobutyric acid
VTA: ventral tegmental area
P: preferring
Ad: adenovirus
LV: lentiviral
CPP: conditioned place preference
